# Unexpected pheochromocytoma presenting as a pancreatic tumor: A case report

**DOI:** 10.3892/ol.2013.1447

**Published:** 2013-07-08

**Authors:** YI-HSUAN HUANG, WEN-JINN LIAW, CHANG-PO KUO, ZHI-FU WU, CHEN-HWAN CHERNG, JYH-CHERNG YU, HUEI-CHI HORNG, SHUN-TSUNG HUANG

**Affiliations:** 1 Department of Anesthesiology, Tri-Service General Hospital and National Defense Medical Center, Taipei 11490, Taiwan, R.O.C.; 2 Department of Surgery, Tri-Service General Hospital and National Defense Medical Center, Taipei 11490, Taiwan, R.O.C.; 3 Department of Anesthesiology, Taichung Armed Forces General Hospital, Taiping, Taichung 41152, Taiwan, R.O.C.

**Keywords:** intra-abdominal tumor, pancreatic tumor, pheochromocytoma

## Abstract

A 54-year-old female presented with a large pancreatic tumor of the tail during a regular physical examination. The patient underwent surgical intervention and the surgeon identified that the tumor originated from the retroperitoneal region. Markedly severe hemodynamic fluctuations occurred during the manipulation of the tumor and continued to occur subsequent to the tumor being removed. The vital signs were adequately managed and the surgery was successful without complications. The patient was discharged without any sequelae days later. The pathology report indicated a diagnosis of pheochromocytoma. Unexpected pheochromocytoma may lead to a fatal hypertensive crisis with catastrophic sequelae during surgery. The peri-operative management of pheochromocytoma remains a complicated challenge that requires intensive pre-operative preparation and vigilant peri-operative care. For surgeons and anesthesiologists who may encounter an unexpected hypertensive crisis during abdominal tumor surgery, undiagnosed pheochromocytoma should always be considered.

## Introduction

Pheochromocytomas occur most frequently in individuals aged 40–50 years, with a slight predilection in females (1). The tumors produce catecholamine, which may lead to severe hypertension and other systemic disturbances (1). Anesthetic management of any surgical patient with pheochromocytoma is challenging and may be difficult to deal with if the tumor has not been diagnosed. A proportion of patients are diagnosed at the time of incidental surgery and, in this situation, the mortality rate is ~80% (2). The serious and potentially lethal nature of this complication is caused by the potent effect of the paroxysmal release of catecholamines. Physicians should be aware of the clinical manifestations and complications of excess catecholamine and be ready to provide proper pre-operative management to minimize catecholamine-related pre-, intra- and post-operative adverse events (3,4). Written informed consent was obtained from the patient.

## Case report

**Figure 1:**
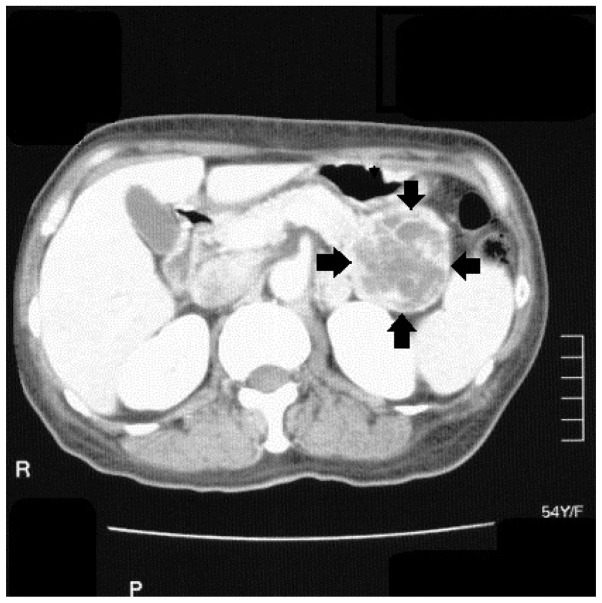
Abdominal computed tomography showing a well-encapsulated tumor located at the tail of the pancreas. (indicated by arrows).

A 54-year-old female (weight, 46 kg; height, 153 cm), was transferred to Tri-Service General Hospital on account of an unexpected large pancreatic tumor. The clinical history of the patient included paroxysmal headaches, mildly elevated blood pressure (BP), diaphoresis and occasional palpitations. The patient was previously diagnosed with ventricular arrhythmia by cardiovascular departments in numerous hospitals, without any other significant findings. The patient was not administered regular treatment for the headaches or hypertension as the symptoms were considered insignificant. One month prior to surgery, the patient underwent a detailed health checkup and an abdominal mass was identified using abdominal sonography. A large, well-encapsulated pancreatic tail tumor, measuring 9 cm in length, was observed on abdominal computed tomography (Fig. 1). The patient was consequently transferred for surgical intervention.

On admission, mildly elevated BP (138–160/80–90 mmHg) with a heart rate (HR) of 70–90 beats per minute (bpm) was noted. The ECG revealed a normal sinus rhythm with two ventricular premature contractions (VPCs). Other laboratory tests showed no significant abnormalities. An exploratory laparotomy with a resection of the tumor was scheduled. Thoracic epidural anesthesia was initially performed without adverse events, followed by general anesthesia. When the pancreas was approached, no any abnormal lesions were identified, with the exception of a bulging mass from the retroperitoneal region. The mass originated from the adrenal gland and presented as a capsulated, vessel-rich tumor. The systolic BP surged to 260 mmHg abruptly with fluctuations and the HR increased to 150 bpm during the manipulation of the tumor. The concentration of the anesthesia was increased along with an additional administration of 100 μg intravenous (i.v.) fentanyl. The fentanyl was ineffective and 5 mg i.v. labetalol was administered twice. However, the hypertensive crisis remained. The surgeon made a temporary stay of surgery until the vital signs were under control and then the tumor was removed.

**Figure 2:**
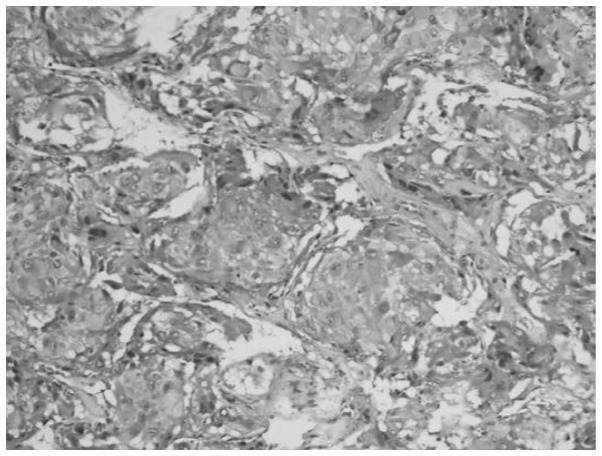
Pathology of the tumor showing a pheochromocytoma. Hematoxylin and eosin staining; magnification, ×400.

The BP dropped (75/50 mmHg) once the tumor was removed. Aggressive fluid replacement and vasopressors were administered until the patient was hemodynamically stable. The endotracheal tube was then removed. At one day post-surgery, the patient was completely asymptomatic and no sequelae were identified. The pathological report confirmed a diagnosis of pheochromocytoma (Fig. 2) and the patient was discharged five days later.

## Discussion

Although the majority of pancreatic tumors are malignant, others, including insulinomas, gastrinomas and vasoactive intestinal peptide-producing tumors (VIPoma), are benign endocrine tumors (5). The most common clinical symptoms are gastrointestinal (GI) tract discomfort, including jaundice, abdominal pain, anorexia and body weight loss (6). These symptoms were not observed in the present patient. Adrenal gland tumors are identified in as many as 10% of autopsies and the majority are asymptomatic (1).

Pheochromocytoma is one of these adrenal tumors and may lead to life-threatening events if precautions are not taken, particularly during surgery. The symptoms and signs that may be solicited are paroxysmal attacks of sweating, headaches, hypertension, glucose intolerance and arrhythmia, which may occur in certain cases (1,7,8). These symptoms and signs were consistent with the patient in the present case. Undiagnosed pheochromocytoma may be catastrophic for physicians, as it accounts for 25–50% of hospital mortalities during the induction of anesthesia or during surgical procedures (9). In the present study, upon reviewing the patient's past history, no GI symptoms were observed, but the palpitations, headaches, diaphoresis and the image presentation of this case mimicked a pancreatic tail tumor (Fig. 1). Misdiagnoses may occur easily, resulting in adverse events. The present case provides first-line clinical physicians with an additional option to consider as a diagnosis when dealing with a suspicious pancreatic tumor. Performing the appropriate history and physical examinations is always the most important diagnostic action. If the initial diagnosis is not consistent with these previously mentioned subjective complaints, a more detailed history or comprehensive examination is required. For anesthesiologists and surgeons who encounter an unexpected hypertensive crisis during abdominal tumor surgery, undiagnosed pheochromocytoma should always be considered as an option.
